# Supplementary figures and images for: Proteomic analysis of differential anther development from sterile/fertile lines in Capsicum annuum L
Source: PeerJ. 2022 May 27;10:e13168. doi: 10.7717/peerj.13168 (PMC9150696; doi:10.7717/peerj.13168)

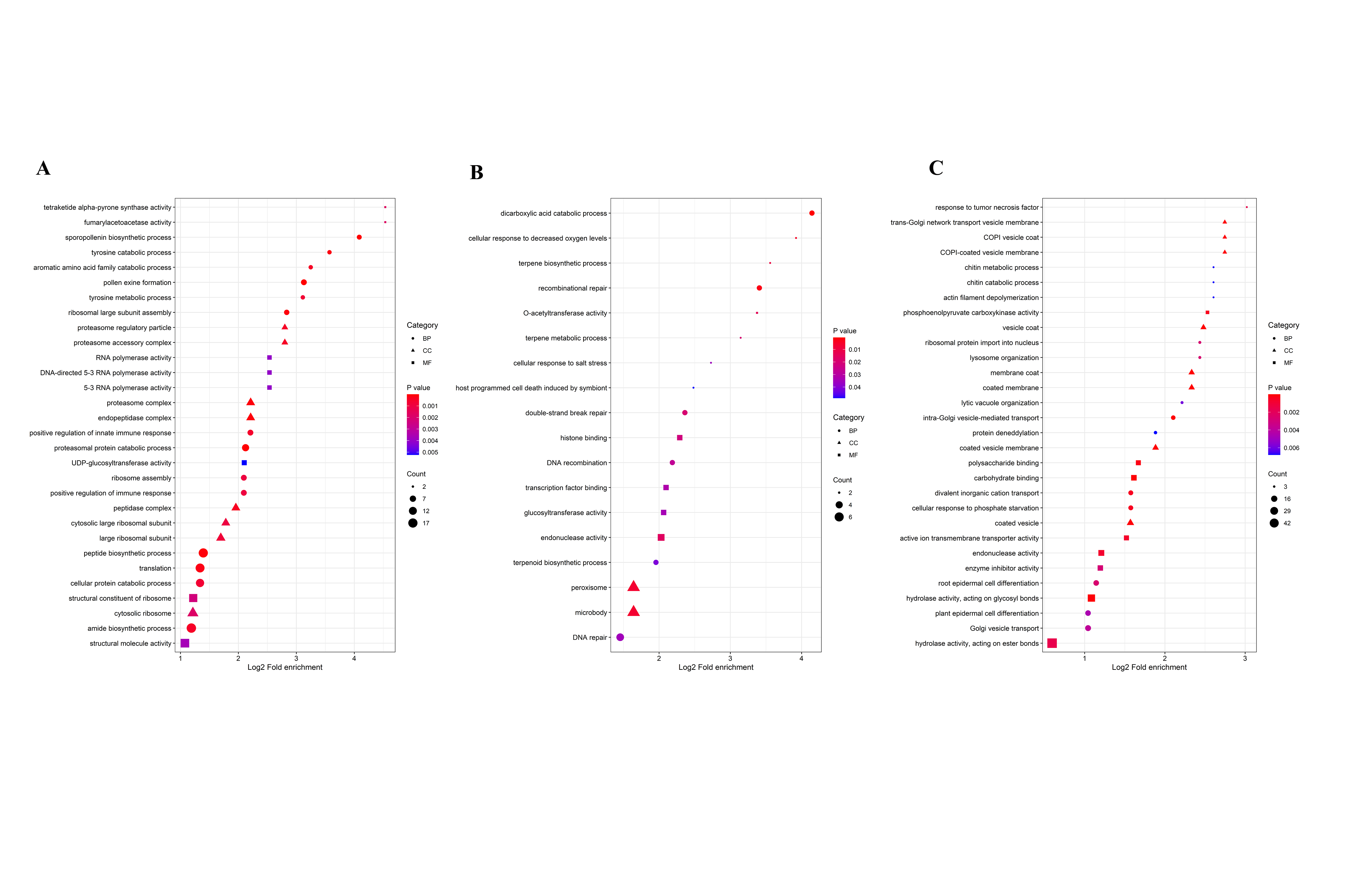

Supplement: Supplemental Information 1 — A: BY_JS vs. KY_JS. B: BY_SF vs. KY_SF. C: BY_SH vs. KY_SH. BP, biological process; MF, molecular function; CC, cellular component. [file peerj-10-13168-s001.tif]

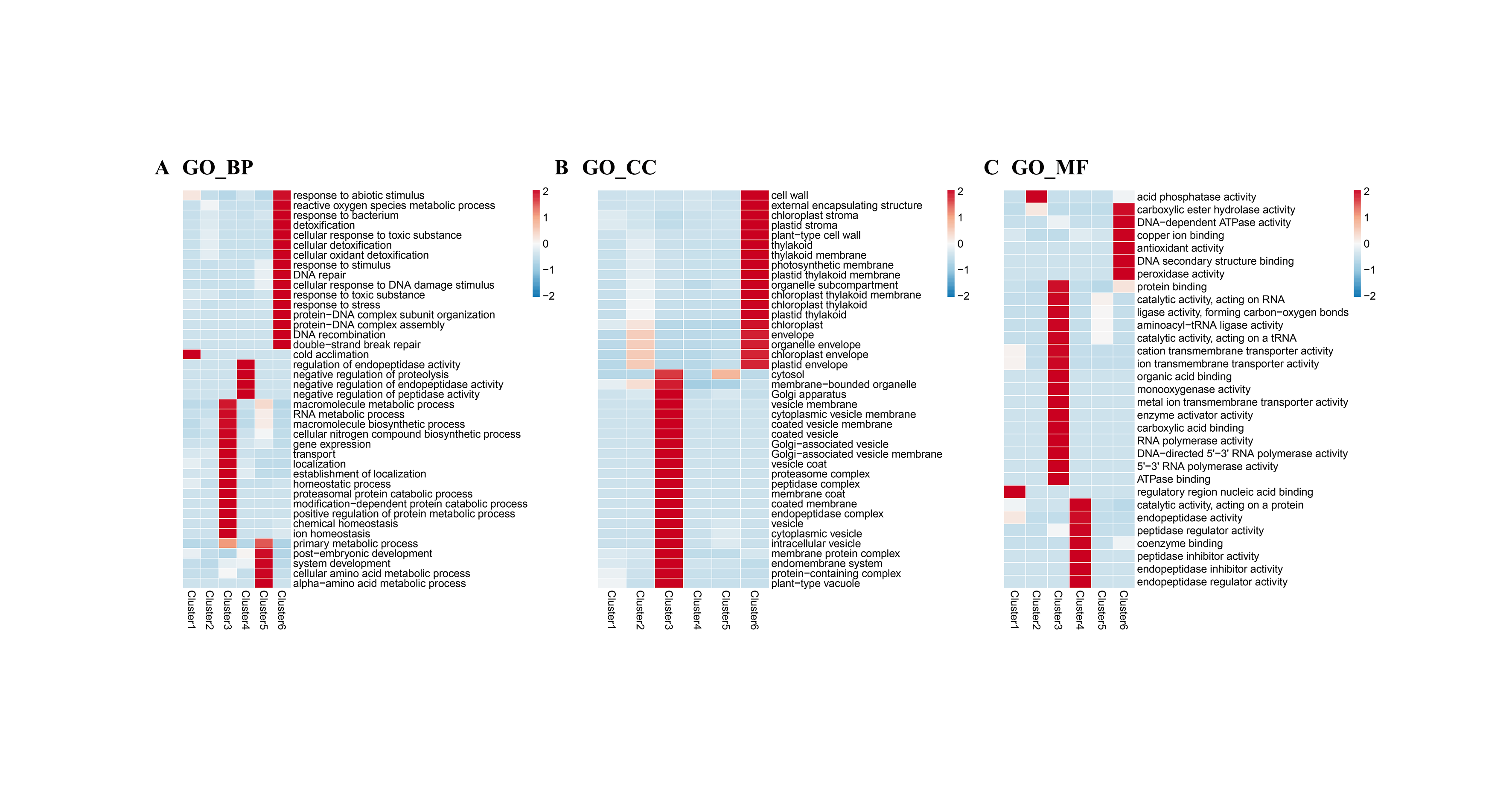

Supplement: Supplemental Information 2 — A: Gene ontology enrichment of DAPs in biological process; A: Gene ontology enrichment of DAPs in cellular component; C: Gene ontology enrichment of DAPs in molecular function. BP, biological process; MF, molecular function; CC, cellular component. [file peerj-10-13168-s002.tif]
